# Validation of Multi-Target Stool DNA Methylation Test for Colorectal Cancer Detection: A Preliminary Analysis

**DOI:** 10.3390/biomedicines14050999

**Published:** 2026-04-27

**Authors:** Khairul Anwar Abdul Rahman, Nabil Mohammad Azmi, Shahrun Niza Abdullah Suhaimi, Zairul Azwan Mohd Azman, Farhana Raduan, Khairul Najmi Muhammad Nawawi, Shamsul Azhar Shah, Geok Chin Tan, Yin Ping Wong, Sayyidi Hamzi Abdul Raub

**Affiliations:** 1 Department of Surgery, Faculty of Medicine, Hospital Canselor Tuanku Muhriz, Universiti Kebangsaan Malaysia, Kuala Lumpur 56000, Malaysia; zairulazwan@gmail.com (Z.A.M.A.); farhana@ppukm.ukm.edu.my (F.R.); 2 Department of Medicine, Faculty of Medicine, Hospital Canselor Tuanku Muhriz, Universiti Kebangsaan Malaysia, Kuala Lumpur 56000, Malaysia; khairulnajmi84@gmail.com; 3 Department of Public Health, Faculty of Medicine, Hospital Canselor Tuanku Muhriz, Universiti Kebangsaan Malaysia, Kuala Lumpur 56000, Malaysia; drsham@ppukm.ukm.edu.my; 4 Department of Pathology, Faculty of Medicine, Hospital Canselor Tuanku Muhriz, Universiti Kebangsaan Malaysia, Kuala Lumpur 56000, Malaysia; tangc@ppukm.ukm.edu.my (G.C.T.); ypwong@hctm.ukm.edu.my (Y.P.W.); 5 Cytogenetics & Molecular Diagnostic Laboratory, Premier Intergrated Labs, Kuala Lumpur 59100, Malaysia; sayyidi.hamzi@premierintegratedlabs.com.my

**Keywords:** colorectal cancer, stool DNA methylation, screening test, diagnostic accuracy, early detection

## Abstract

Colorectal cancer (CRC) develops gradually from precancerous adenomas and is highly curable when detected early. In Malaysia, however, most cases are diagnosed at advanced stages, leading to poorer outcomes despite the availability of screening programmes such as the immunological Faecal Occult Blood Test (iFOBT). Limited screening uptake and poor adherence contribute to delayed diagnosis. Therefore, effective non-invasive and patient-friendly screening tools are essential. **Background/Objectives**: This study aims to validate the diagnostic performance of the multi-target stool DNA (mt-sDNA) test. **Methods**: This cross-sectional validation study was conducted at the Endoscopic Center, Hospital Canselor Tuanku Muhriz (HCTM), from January 2024 to September 2025. Adults aged 18–75 years undergoing elective or emergency colonoscopy were included. **Results**: Among 246 patients, most were male (56.5%), Malay (65.2%), and aged 55–75 years (91.3%). CRC prevalence was 8.5%. A significant association was observed between age group and colonoscopy findings (*p* = 0.005), with older individuals more likely to have CRC or adenomatous lesions. Most CRC (90.4%) and advanced adenoma (84.6%) cases occurred in symptomatic patients; however, this difference did not demonstrate a significant association with colonoscopy outcomes (*p* = 0.069). Per rectal bleeding, constitutional symptoms, altered bowel habit, abdominal pain and constipation were significantly associated with CRC and adenomatous lesions. The mt-sDNA test showed a sensitivity of 63.2%, specificity of 85.0%, positive predictive value of 36.4%, and negative predictive value of 94.4%. **Conclusions**: Preliminary findings indicate that mt-sDNA demonstrates good specificity and high negative predictive value, but moderate sensitivity and low positive predictive value.

## 1. Introduction

Most colorectal cancer (CRC) starts from a precancerous growth called a polyp or adenoma. These adenomas are usually asymptomatic and slow-growing, and most of them do not progress to cancer [1]. However, as the adenoma grows, it is more likely to become cancerous [2]. Because the precancerous growth phase is slow and can be detected much earlier, screening for CRC is recommended to reduce the future disease burden [3].

Patients diagnosed with early-stage CRC have a 5-year survival rate of 90% compared to only a 10% survival rate for end-stage CRC [4]. Nevertheless, although preventable, only 40% of CRCs are diagnosed at an early stage. Currently, multiple screening modalities are available to identify precancerous lesions and early-stage CRC [5]. The standard screening options recommended by the United States Preventive Services Task Force (USPSTF), starting at age 50 years for early detection of CRC, include faecal tests, radiographic tests, and scopes for lower gastrointestinal tract visualisation [3].

In Malaysia, CRC is the second most common cancer and contributed to 14.1% of all new cancer cases diagnosed in 2017–2021 [6]. According to the Malaysia National Cancer Registry (MNCR) 2017–2021, CRC is the most common cancer among males (17.5%) and the second most common among females (11.3%) in Malaysia. The incidence increased with age and was slightly higher in males (18.8/100,000) than in females (13.7/100,000) [6].

The CRC screening programme using immunological faecal occult blood test (iFOBT) followed by colonoscopy targeting asymptomatic men and women aged 50–75 was initiated in 2014 with 2-year screening intervals. Due to limited resources, the programme is implemented in phases. The number of people screened by health clinics was less than 1% of the country’s total eligible population annually [7]. Out of those screened, only 60% of the positive iFOBT cases referred for colonoscopy underwent the procedure.

Around 70% of CRC patients in Malaysia were diagnosed at stage III or IV. This is the stage at which treatment is more complicated and the outcome is poorer [6]. The stage of cancer at diagnosis plays an important role in determining the treatment and possible survival. It is noteworthy that CRC is one of the cancers that is highly preventable and treatable through early detection.

The stage at diagnosis is probably the most important determinant of survival. According to the Malaysian Study on Cancer Survival (MySCan), the overall 5-year relative survival for CRC was 51.1% [8]. Improving the stage at diagnosis is achievable by early detection of cancer, and CRC is the type of cancer that can be detected early via screening and early diagnosis.

Many randomised controlled trials have reported a consistent reduction in CRC mortality due to screening [9,10]. This is attributed to early detection and removal of precancerous lesions [10,11]. However, despite the well-established association between the screening test and reduced CRC mortality, its preventive effectiveness may be diminished due to poor adherence, which is further associated with test invasiveness [12]. The non-invasive tests are, therefore, specifically designed to consider patient preference, compliance, and ease of use [12]. The American Cancer Society (ACS) also notes that these non-invasive tests are more appealing to patients than colonoscopy [12].

The American Cancer Society (ACS) recommends several colorectal cancer (CRC) screening modalities, including stool-based tests such as the faecal immunochemical test (FIT), guaiac-based faecal occult blood test (gFOBT), and multitarget stool DNA test, as well as structural examinations such as colonoscopy, computed tomographic (CT) colonography, and flexible sigmoidoscopy [12].

gFOBT uses a chemical to detect heme, while FIT uses antibodies to specifically detect haemoglobin. In comparison, the stool DNA test detects haemoglobin, along with specific DNA biomarkers. Among stool-based tests, FIT demonstrates a sensitivity of approximately 73–88% and specificity of 90–96% for CRC detection [12,13], whereas gFOBT has a lower sensitivity of approximately 50–75% but maintains high specificity of 96–98% [13]. Multitarget stool DNA testing has a sensitivity of approximately 92.3% for CRC, with a specificity of about 86.6% [14]. However, it is recommended that the stool DNA test should be repeated every 1 to 3 years [15]. Hence, this study aims to validate the capability of the multi-target stool DNA (mt-sDNA) test for the early detect CRC early.

Colonoscopy remains the reference standard, with a reported sensitivity of approximately 89–98% for adenomas ≥10 mm [11,16], due to its ability to directly visualise and remove precancerous lesions. Nonetheless, compliance and accessibility issues limit its feasibility as a population-wide screening tool, especially in resource-constrained settings [3,17,18]. This limitation has prompted the development of non-invasive molecular tests, such as stool DNA methylation assays, which can detect early epigenetic alterations associated with tumorigenesis [19,20,21,22].

The primary study outcome is to assess the diagnostic performance of the mt-sDNA Colorectal Cancer Screening Test in a colonoscopy-referred population, as a preliminary validation prior to a population-based screening study. The secondary objectives were to describe the characteristics of the study population (sociodemographic and clinical characteristics), to determine the prevalence of CRC among patients undergoing colonoscopy, and to evaluate the sensitivity, specificity, positive predictive value, and negative predictive value of the mt-sDNA Colorectal Cancer Screening Test.

## 2. Materials and Methods

This is an international collaborative study among Universiti Kebangsaan Malaysia (UKM), Yaneng BIOsciences (Shenzhen) Co., Ltd., (Shenzen, China) and Premier Integrated Labs Sdn. Bhd. (Kuala Lumpur, Malaysia) This was a prospective diagnostic accuracy study with cross-sectional analysis, conducted among patients undergoing colonoscopy at the Endoscopy Centre Hospital, Canselor Tuanku Muhriz (HCTM). The study was conducted from 4 September 2023 to 3 September 2025.

### 2.1. Patient Recruitment

**Figure 1:**
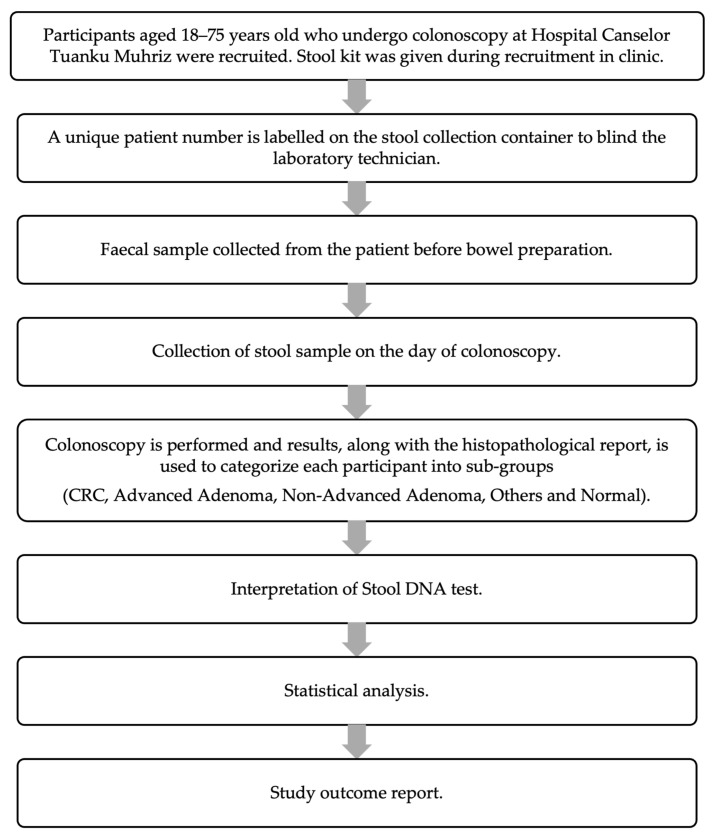
Study process of recruitment of patient until analysis.

Patients were recruited before the endoscopy procedure in the surgical clinic or wards at HCTM using convenience sampling. Written consent was taken in the clinic during recruitment in accordance with the principles outlined in the Declaration of Helsinki (1975, revised in 2013). A proforma was completed to collect the demographic data of the patients along with clinical data of CRC risk factors, symptoms, and indications for colonoscopy. The flow of the recruitment process is shown in Figure 1.

The risk factors include smoking, alcohol consumption, weight, height, and body mass index (BMI). Smokers were defined as patients with a history of smoking (including both current and former smoking), and non-smokers were defined as those with no history of smoking. Alcohol consumption was defined as drinkers (individuals with any history of alcohol intake) and non-drinkers (those who had never consumed alcohol). BMI were grouped into 6 groups which are Underweight (<18.5 kg/m^2^), Normal (18.5–22.9 kg/m^2^), Overweight (23–27.4 kg/m^2^), Obese class I (27.5–32.4 kg/m^2^), Obese class II (32.5–37.4 kg/m^2^) and Obese class III (>37.5 kg/m^2^) [23].

**Figure 2:**
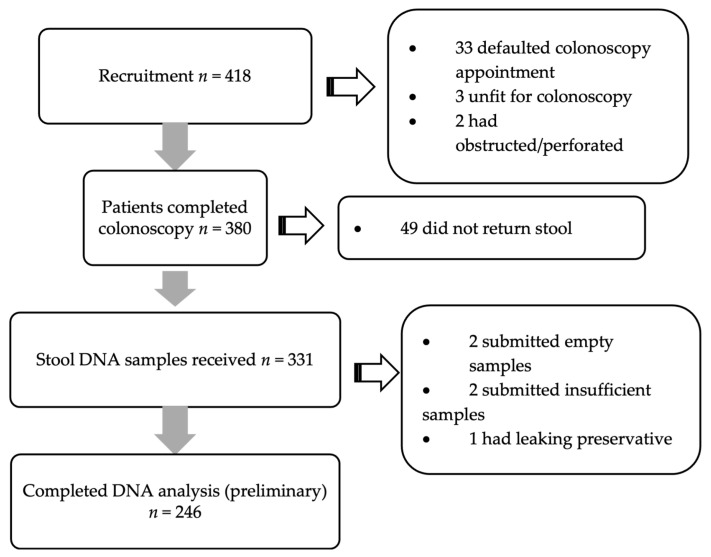
Study process of recruitment of patient until preliminary analysis. Only 246 DNA analysis was done due to financial realignment by the funding company.

The study comprised patients aged 18 to 75 years who were recruited for colonoscopy (both elective and emergency). The exclusion criteria were those with known CRC and who have undergone resection surgery, chemotherapy, or radiotherapy; patients with known polyps/adenomas that have been removed; and patients with loose or watery stool. Figure 2 shows the total number of patients recruited during this preliminary analysis.

### 2.2. Stool Sample Collection

The patients collected stool samples before bowel preparation and handed them in during the colonoscopy. The stool samples were then collected and sent to a third-party lab (Premier Integrated Labs Sdn. Bhd), who was assigned by Yaneng Bioscience (Shenzhen, China) Co. Ltd. The samples were stored in a −40 °C refrigerator. The laboratory staff were blinded to protect anonymity of the patients and to prevent analysis bias. Results of the multi-target stool DNA testing were grouped by pathological features observed during colonoscopy, with histopathological confirmation serving as the reference standard for diagnostic performance analysis. Patients were then grouped into five categories based on their colonoscopy and histopathological results (CRC, Advanced Adenoma, Non-Advanced Adenoma, Others, and Normal). An Advanced Adenoma is classified as a polyp that is 1 cm or more, exhibits villous/tubulovillous features, or has high-grade dysplasia [1]. Other diagnoses on colonoscopy that do not belong to other groups (e.g., colitis, haemorrhoids) were put in the “Others” group.

### 2.3. Sample Treatment

Sample treatment and DNA analysis were done in certified private diagnostic laboratory. DNA was isolated using the Nucleic Acid Extraction Reagent (Yaneng BIOsciences). Bisulfite treatment and DNA purification were done using the Pretreatment Kit for Methylation Detection (Yaneng BIOsciences). The conversion of genomic DNA using bisulfite was first introduced by Frommer et al. and considered a gold standard for the detection of DNA methylation [24]. The extracted and transformed stool DNA was tested using quantitative methylation-specific PCR (qMSP).

### 2.4. Detection of Methylated Gene in Samples

The genes Syndecan 2 (SDC2), Septin 9 (SEPT9), and Tissue Factor Pathway Inhibitor 2 (TFPI2) were selected as targets for DNA methylation analysis due to their well-established roles as epigenetic biomarkers in colorectal cancer (CRC). Aberrant methylation of these genes has been consistently reported in CRC tissues and stool samples, with demonstrated utility in early detection. Specifically, SEPT9 methylation is one of the most extensively validated blood-based biomarkers for CRC screening, while SDC2 and TFPI2 methylation have shown high sensitivity and specificity in stool-based detection of colorectal neoplasia [19,20,21,25,26]. Real-time PCR amplification was performed using the SLAN Real-Time PCR System (Yaneng Bio) to detect stool-based DNA methylation in the SDC2, SEPTIN9, and TFPI2 genes.

The ACTB (β-actin) gene was used as an internal control to assess DNA integrity and adequacy of sample input. As a constitutively expressed housekeeping gene, ACTB serves as a normalisation reference and ensures amplification reliability in PCR-based methylation assays [24]. This study was blinded to eliminate the influence of known colonoscopy results. Microsoft Excel was used to assign numbers to the collected stool samples, while their original tracing numbers were removed.

### 2.5. Data Analysis

Data analysis was performed using the Statistical Package for the Social Sciences (SPSS) version 31. Descriptive statistics were used to describe the demographic profile. The normality of the continuous data was determined using a histogram with a normal curve. The association between categorical variables and study groups was determined using a Chi-square test or Fisher’s exact test, as appropriate. Fisher’s exact test was used when the expected cell counts were less than five. Monte Carlo simulation was used where appropriate to estimate *p*-values. A preliminary analysis was conducted to assess the diagnostic performance of the mt-sDNA test before proceeding with a full validity study. Invalid mt-sDNA results were excluded from diagnostic performance calculations. Stool DNA analysis was discontinued earlier than planned following the termination of project sponsorship. Diagnostic performance was evaluated using sensitivity, specificity, positive predictive value (PPV), and negative predictive value (NPV).

Sensitivity = (True Positives/(True Positives + False Negatives))(1)

Specificity = (True Negatives/(True Negatives + False Positives))(2)

Positive predictive value (PPV) = (True Positives/(True Positives + False Positives))(3)

Negative predictive value (PPV) = (True Negatives/(True Negatives + False Negatives))(4)

Patients with colorectal cancer were excluded from the analysis of advanced and non-advanced adenoma diagnostic performance, as the objective was to evaluate the test’s ability to detect precancerous lesions. Inclusion of CRC cases would overestimate sensitivity due to higher biomarker expression and would not reflect true adenoma detection performance.

### 2.6. Ethical Consideration

This study was conducted at Hospital Canselor Tuanku Muhriz (HCTM), Universiti Kebangsaan Malaysia (UKM) and was approved by the research and ethics committee Universiti Kebangsaan Malaysia (RECUKM) (JEP-2023-516) on 11 September 2023. All participants provided written informed consent before enrolment. The data was anonymized and entered into a password-protected database for subsequent analysis. The documents were stored in the designated research room at the Central Hospital (HCTM). The stool samples were collected, sent to a third-party lab (Premier Integrated Labs Sdn. Bhd.), and destroyed 3 months after data analysis was completed. The anonymity of the stool samples was protected using number labelling, which does not allow identification of the source.

## 3. Results

The study sample that had undergone a complete colonoscopy comprised 380 participants (201 females, 52.8%, and 179 males, 47.1%), indicating a slight female predominance. Regarding ethnicity, the majority were Malay (51.8%), followed by Chinese (41.3%), Indian (5.5%), and others (1.3%). Most patients recruited were 55–75 years old (67.6%), followed by 45–54 (13.4%), 35–44 (11.0%), and 18–34 (7.8%). Chi-square and Fisher exact tests showed significant associations between colonoscopy findings and gender (*p* = 0.040), ethnicity (*p* = 0.022), and age group (*p* < 0.001), as shown in Table 1, indicating that older age was significantly associated with abnormal colonoscopy findings, particularly CRC and adenomas. The prevalence of CRC in the whole cohort was 10.5% (40/380 patients).

### 3.1. Preliminary Analysis

In the preliminary analysis, among participants who underwent colonoscopy (N = 246), males had a higher proportion of CRC (12.8%) than females (8.5%). By ethnicity, Malay participants had the highest proportion of CRC (14.2%), followed by Chinese (6.4%) and Indian (4.8%). The “Other” ethnicity had a CRC proportion of about 20%, but this was partly due to the small sample size. CRC and adenomatous lesions were predominantly found in the 55–75 age group (91.3%), confirming a strong age-related trend. The Chi-square and Fisher exact analysis revealed no significant association between colonoscopy findings and gender (*p* = 0.053) or ethnicity (*p* = 0.261), but a significant association with age group (*p* = 0.005), as shown in Table 2, indicating older individuals were more likely to have CRC and adenomas. This result is consistent and shows that the preliminary analysis group has demographics similar to those of the rest of the sample. The prevalence of CRC in the preliminary analysis cohort was 8.5% (21/246).

### 3.2. Risk Factor of CRC

Meanwhile, for the risk factors associated with colorectal carcinoma, smokers had a CRC rate of 11.1%, similar to ex-smokers (16.7%), while non-smokers had a slightly lower CRC rate (7.7%). Alcohol consumption did not contribute any proportion towards CRC due to the low number of samples. Non-drinkers had a CRC proportion of 9.1%. In terms of BMI, most CRC cases occurred among overweight individuals (59.1%), followed by those with normal BMI (22.7%), while obesity was less common. These findings suggest that CRC occurrence was higher in overweight, non-smoking, and non-drinking individuals, with limited influence from family history or obesity. Overall, the exact Fisher analysis showed no significant association between colonoscopy findings and smoking status (*p* = 0.363), drinking habits (*p* = 0.670), family history of CRC (*p* = 0.087), or BMI group (*p* = 0.136) as shown in Table 3.

### 3.3. Symptoms of CRC

In the symptom category, 186 (75.6%) were symptomatic and 60 (24.4%) were asymptomatic. Symptomatic patients demonstrated a higher proportion of clinically significant pathology, particularly colorectal cancer (10.3%) and advanced adenomas (5.9%). In contrast, among asymptomatic individuals, the proportion of CRC was markedly lower at 3.3%, with advanced adenoma also at 3.3%, and non-advanced adenoma slightly higher at 26.2%; “others” accounted for 16.4%, and a substantially higher proportion (50.8%) had normal findings.However, the difference did not reach statistical significance (*p* = 0.069), indicating only a trend toward association as shown in Table 4. Among patients with colonoscopy findings, per rectal (PR) bleeding (52.3%) and altered bowel habits (42.8%) were the most common symptoms among CRC cases. Overall, PR bleeding emerged as the most prominent presenting feature associated with CRC and adenomatous lesions, highlighting its diagnostic relevance in colorectal screening. The exact Fisher analysis showed significant associations between colonoscopy findings and the symptoms PR bleeding (*p* < 0.001), constitutional symptoms (*p* ≤0.001), altered bowel habit (*p* = 0.03), abdominal pain (*p* = 0.006), and constipation (*p* = 0.003) as shown in Table 5.

### 3.4. Multitarget Stool DNA (mt-sDNA) Results

For the preliminary analysis, DNA from stool was analysed in 246 stool samples. Of the 246 samples, 33 (13.4%) had positive DNA methylation results, 126 (51.2%) had negative results, and 87 (35.3%) had invalid results. Among individuals with a positive mt-sDNA result, 36.4% were diagnosed with colorectal cancer (CRC), while 9.1% had advanced adenoma, 15.2% had non-advanced adenoma, 15.2% had other findings, and 24.2% had normal colonoscopy findings. In contrast, among those with a negative mt-sDNA result, the majority had normal findings (38.9%), followed by other findings (25.4%) and non-advanced adenoma (24.6%), with only 5.6% having CRC and 5.6% having advanced adenoma. Similarly, individuals with invalid results predominantly had normal findings (44.8%), with lower proportions of CRC (2.3%) and advanced adenoma (3.4%). These findings indicate that a positive mt-sDNA result is associated with a higher proportion of clinically significant pathology, particularly CRC, whereas negative and invalid results are more frequently associated with normal or less significant findings. The performance of the multitarget stool DNA test demonstrated a statistically significant association with colonoscopy findings (*p* < 0.001) as shown in Table 6.

Among those with colorectal cancer (CRC), 12 individuals tested positive while 7 tested negative. In the non-CRC group, 21 individuals had a positive test result and 119 tested negative as shown in Table 7. The mt-sDNA test demonstrated the highest positivity among CRC patients (57.1%), with a sensitivity of 63.2%, a specificity of 85%, a positive predictive value (PPV) of 36.4%, and a negative predictive value (NPV) of 94.4% as shown in Table 8. These findings highlight the mt-sDNA test’s strong potential as a non-invasive screening tool with excellent rule-out capability for CRC detection in this preliminary analysis.

The diagnostic performance of multitarget stool DNA (mt-sDNA) testing for the detection of advanced adenomas (AA) is summarised in Table 9. CRC was excluded from non-AA because it is a more advanced disease than AA. When analysed against colonoscopy findings as the reference standard, the mt-sDNA test achieved a sensitivity of 30.0%, specificity of 86.2%, PPV of 14.3%, and NPV of 94.1% for detecting advanced adenomas as shown in Table 10. The diagnostic performance of multitarget stool DNA (mt-sDNA) testing for the detection of non-advanced adenomas (NAA) is summarised in Table 11. For non-advanced adenomas, the test showed a sensitivity of 13.9%, specificity of 84.6%, PPV of 23.8%, and NPV of 74.0% as shown in Table 12.

Overall, the mt-sDNA test showed a clear trend of higher positivity in CRC compared to the other groups, supporting its diagnostic value for CRC detection; however, invalid and negative rates remain notable across non-cancer groups.

## 4. Discussion

In this preliminary analysis of 246 patients, the demographic profile was consistent with national trends reported by the Malaysia National Cancer Registry, showing a higher proportion of males, Malay ethnicity, and a predominance in the 55–75-year age group [6]. The significant association between age and abnormal colonoscopy findings reinforces established evidence that the risk of colorectal neoplasia increases with age [4,6]. This supports current screening recommendations by the American Cancer Society (2017), which advocate for population-based screening beginning at age 50, or earlier in high-risk groups, to enable early detection and reduce disease burden [12].

Symptomatic presentation was also significantly related to colonoscopy findings, with most cases of CRC and advanced adenoma identified among symptomatic individuals. This aligns with earlier studies demonstrating that symptoms such as PR bleeding and altered bowel habits often correlate with advanced disease at presentation [3]. The significant association between per rectal bleeding, tenesmus, and constipation with colorectal cancer or adenomatous lesions underscores the importance of prompt endoscopic evaluation in symptomatic patients, consistent with previously reported diagnostic patterns [9,15]. Odds ratios were not reported for certain variables due to small cell counts, which may result in unstable estimates and wide confidence intervals.

The relatively high number of invalid mt-sDNA results in this preliminary analysis highlights several practical challenges that need to be resolved before the test can be implemented on a larger scale. Stool-based DNA assays are particularly vulnerable to pre-analytical factors such as inadequate sample volume, variable stool consistency, DNA degradation, and issues with sample preservation, all of which can affect test reliability [22,27]. Notably, large prospective studies have reported a proportion of non-evaluable stool DNA samples due to inadequate DNA quantity or assay failure, suggesting that invalid results may represent a recognised limitation of the testing approach rather than a study-specific occurrence [22]. In routine clinical settings, patient-related factors—including difficulties with stool collection and altered bowel habits in symptomatic individuals—may further increase the likelihood of unusable samples. In addition, logistical issues, such as sample transport and differences in laboratory workflows, can affect assay performance. These findings suggest that the observed invalid results primarily reflect real-world implementation challenges, underscoring the importance of improved collection protocols, patient education, and laboratory standardisation to support broader clinical use.

In this study, the mt-sDNA test demonstrated a sensitivity of 63.2% and specificity of 85.0% for colorectal cancer. Although higher sensitivities exceeding 80% have been reported for certain multi-marker stool DNA assays in previous investigations [14], differences in assay composition, study design, and population characteristics may account for the variation observed. The observed negative predictive value (NPV) of 94.4% is particularly reassuring, suggesting that a negative mt-sDNA result effectively excludes colorectal cancer. This supports its potential application as an adjunctive screening tool to colonoscopy, in line with prior reports showing high NPV and specificity for stool-based DNA methylation biomarkers such as SDC2 and BMP3 [19,20,28,29].

The moderate positive predictive value (PPV) of 36.4% observed in this study may be explained by the inclusion of early-stage or small lesions associated with limited DNA shedding, as well as potential sample degradation during processing, a limitation previously reported in the literature [22,27]. Nevertheless, the mt-sDNA assay demonstrates greater sensitivity than immunochemical faecal occult blood testing (iFOBT), particularly for adenomatous and right-sided lesions [14,30]. In addition, the non-invasive nature of the test may enhance patient compliance and convenience, thereby helping to address barriers to widespread screening uptake [5,12].

The mt-sDNA assay detects methylated DNA markers shed by neoplastic cells into stool, reflecting early molecular alterations along the adenoma–carcinoma sequence [26]. The combination of multiple markers, as in mt-sDNA panels, improves diagnostic accuracy by capturing a wider range of molecular changes [14,31].

The relatively lower sensitivity observed in this Malaysian cohort may be influenced by population-specific genetic and epigenetic variability, as previously suggested [4], and warrants further validation in larger, multicentre studies. Environmental and dietary factors, including fibre intake and red meat consumption, may also modulate colorectal carcinogenesis and associated methylation patterns [8]. In addition, pre-analytical variables such as sample degradation and handling conditions can affect the detection of methylated DNA targets, underscoring the importance of standardised stool collection and processing protocols [27].

The mt-sDNA showed lower sensitivity in detecting advanced and non-advanced adenomas compared with CRC screening. However, it still has comparative specificity and negative predictive value for advanced adenoma (86.2% and 94.1%) and non-advanced adenoma (84.6% and 74.0%).

These findings indicate that while mt-sDNA demonstrates strong specificity and high negative predictive value—suggesting its potential reliability in ruling out advanced lesions—it has comparatively lower sensitivity for adenomatous changes, particularly in non-advanced adenomas. Similar trends have been reported in earlier studies, where sensitivity for adenomas is generally lower than for colorectal carcinoma due to reduced levels of aberrant methylation and DNA shedding in early lesions [19,21,29].

Overall, this study demonstrates that the mt-sDNA test offers a promising, non-invasive adjunct to colonoscopy and is particularly valuable for population-level screening and patients who are reluctant or unsuitable for endoscopic procedures. Its high NPV supports its role as an effective rule-out test. Simultaneously, its moderate sensitivity highlights the need for combined screening strategies, integrating molecular stool testing with colonoscopy follow-up for positive cases. This approach aligns with global trends toward multi-modality screening programmes aimed at improving early CRC detection and survival outcomes [11,12,14].

### 4.1. Limitations

This study contributes important evidence on the relationship between demographic characteristics, clinical features, and early molecular detection markers of CRC and advanced adenomas in a Malaysian population. However, several methodological and logistical limitations should be taken into consideration when interpreting these findings.

#### 4.1.1. High Rate of Invalid Stool DNA Samples

Although the study design anticipated an approximate 10% dropout rate due to potential participant noncompliance or stool collection challenges, the actual proportion of non-analysable samples was higher than expected. After recruitment, only 331 stool samples were submitted, with 33 of 418 (7.89%) patients defaulting from the colonoscopy appointment. In total, 5 were unfit for the colonoscopy (1.19%). Another 49 patients (11.7%) came for colonoscopy but did not submit their samples due to various reasons, such as difficulty handling the stool collection, loose stool, no stool output during the requested period of collection, and refusal to participate in the study. This contributes to the dropout rate (20.8%), which is higher than expected. During preliminary analysis, 35.3% specimens analysed were deemed invalid during molecular testing. This may have resulted from i) technical issues, such as sample viscosity affecting DNA extraction and sample degradation, or ii) biological factors, particularly in participants with normal mucosa and minimal DNA shedding. The dropout of these patients should be understood in two ways: it reflects challenges in the stool sample testing process (laboratory issues) as well as natural differences and irregularities among the patients themselves.

#### 4.1.2. Variability Between Initial and Final Pathological Diagnoses

A small subset of cases demonstrated discrepancies between the initial colonoscopy pathology and the final histopathological diagnosis following surgical resection. Such inconsistencies, though uncommon, are recognised in clinical practice and may arise from sampling limitations, lesion heterogeneity, or inter-observer variability among pathologists. Therefore, histopathological findings should be integrated with clinical and endoscopic assessments rather than interpreted in isolation to ensure diagnostic precision.

#### 4.1.3. Limited Generalisability to the National Population

The study cohort was recruited exclusively from a single tertiary hospital located in the Klang Valley, which may not fully represent Malaysia’s overall population profile. Socioeconomic differences, dietary practices, and healthcare accessibility vary considerably between urban and rural regions, potentially influencing both disease prevalence and screening uptake. Hence, future studies should adopt a multicentric recruitment approach, covering both urban and rural healthcare facilities across different states, to enhance the representation of the whole Malaysian population.

## 5. Conclusions

This study demonstrates that colorectal cancer (CRC) and adenomatous lesions are significantly associated with increasing age and the presence of symptoms, particularly constipation, tenesmus, and per rectal (PR) bleeding. The findings highlight the importance of early screening and timely colonoscopy evaluation in symptomatic or high-risk individuals.

In this preliminary study, the multi-target stool DNA (mt-sDNA) test showed moderate diagnostic sensitivity and high specificity with an excellent negative predictive value, indicating its reliability as a non-invasive tool for ruling out CRC.

The mt-sDNA assay’s performance in this Malaysian cohort supports its potential as a complementary non-invasive test, particularly in resource-limited settings where access to colonoscopy and compliance are constrained. The incorporation of methylation-based stool DNA testing could enhance screening uptake by offering a simple, patient-friendly alternative that could supplement existing methods’ stool-based screening tests.

Future research should focus on expanding sample size, validating marker combinations in local populations, and standardising stool collection and processing protocols to minimise invalid results. Follow-up studies are also warranted to evaluate mt-sDNA performance in detecting post-treatment recurrence.

In summary, this study provides preliminary evidence that mt-sDNA testing is a promising, non-invasive adjunct for early CRC screening, capable of improving detection and reducing disease burden when used alongside conventional screening modalities.

## Figures and Tables

**Table 1:** Demographic data of the patient recruited with complete colonoscopy done (*n* = 380).

		CRC		Advanced Adenoma		Non-Advanced Adenoma		Others		Normal		Total	*p* Value
		Count	Row %	Count	Row %	Count	Row %	Count	Row %	Count	Row %	Count	
Gender	Male	23	12.8%	5	2.8%	51	28.5%	46	25.7%	54	30.2%	179	0.040 *
	Female	17	8.5%	10	5.0%	39	19.4%	50	24.9%	85	42.3%	201	
Ethnicity	Malay	28	14.2%	7	3.6%	35	17.8%	56	28.4%	71	36.0%	197	0.022 ^#^
	Chinese	10	6.4%	7	4.5%	46	29.3%	37	23.6%	57	36.3%	157	
	Indian	1	4.8%	1	4.8%	9	42.9%	1	4.8%	9	42.9%	21	
	Others	1	20.0%	0	0.0%	0	0.0%	2	40.0%	2	40.0%	5	
Age Group	55–75	34	13.2%	13	5.1%	76	29.6%	60	23.3%	74	28.8%	257	<0.001 ^#^
	45–54	4	7.8%	1	2.0%	4	7.8%	12	23.5%	30	58.8%	51	
	35–44	2	4.8%	1	2.4%	6	14.3%	17	40.5%	16	38.1%	42	
	18–34	0	0.0%	0	0.0%	4	13.3%	7	23.3%	19	63.3%	30	
	Total	40	10.5%	15	3.9%	90	23.7%	96	25.3%	139	36.6%	380	

* The Chi-square test was used. ^#^ Fisher’s exact test was used.

**Table 2:** Demographic data of preliminary analysis group (*n* = 246).

		CRC		Advanced Adenoma		Non-Advanced Adenoma		Others		Normal		Total	*p* Value
		Count	Row %	Count	Row %	Count	Row %	Count	Row %	Count	Row %	Count	
Gender	Male	13	10.4%	4	3.2%	34	27.2%	34	27.2%	40	32.0%	125	0.053 *
	Female	8	6.6%	9	7.4%	22	18.2%	26	21.5%	56	46.3%	121	
Ethnicity	Malay	14	11.5%	6	4.9%	20	16.4%	35	28.7%	47	38.5%	122	0.261 ^#^
	Chinese	7	6.4%	6	5.5%	31	28.4%	23	21.1%	42	38.5%	109	
	Indian	0	0.0%	1	7.7%	5	38.5%	1	7.7%	6	46.2%	13	
	Others	0	0.0%	0	0.0%	0	0.0%	1	50.0%	1	50.0%	2	
Age Group	55–75	19	10.7%	12	6.7%	50	28.1%	39	21.9%	58	32.6%	178	0.005 ^#^
	45–54	2	6.7%	1	3.3%	1	3.3%	7	23.3%	19	63.3%	30	
	35–44	0	0.0%	0	0.0%	3	13.6%	10	45.5%	9	40.9%	22	
	18–34	0	0.0%	0	0.0%	2	12.5%	4	25.0%	10	62.5%	16	
	Total	21	8.5%	13	5.3%	56	22.8%	60	24.4%	96	39.0%	246	

* The Chi-square test was used. ^#^ Fisher’s exact test was used.

**Table 3:** Risk factors of colorectal cancer (*n* = 246).

		CRC		Advanced Adenoma		Non-Advanced Adenoma		Others		Normal		Total	*p* Value
		Count	Row %	Count	Row %	Count	Total	Count	Row %	Count	Row %	Count	
Smoking	Yes	3	11.1%	0	0.0%	Total	37.0%	4	14.8%	10	37.0%	27	0.363 ^#^
	Ex-smoker	2	16.7%	0	0.0%	Total	25.0%	4	33.3%	3	25.0%	12	
	No	16	7.7%	13	6.3%	Total	20.8%	52	25.1%	83	40.1%	207	
Drinking	Yes	0	0.0%	0	0.0%	Total	31.3%	5	31.3%	6	37.5%	16	0.670 ^#^
	No	21	9.1%	13	5.7%	Total	22.2%	55	23.9%	90	39.1%	230	
Family history of CRC	Yes	0	0.0%	2	4.3%	Total	30.4%	10	21.7%	20	43.5%	46	0.087 ^#^
	No	21	10.5%	11	5.5%	Total	21.0%	50	25.0%	76	38.0%	200	
BMI group	Underweight	2	14.3%	0	0.0%	Total	14.3%	2	14.3%	8	57.1%	14	0.136 ^#^
	Normal	4	5.3%	3	3.9%	Total	17.1%	21	27.6%	35	46.1%	76	
	Overweight	13	14.3%	6	6.6%	Total	28.6%	19	20.9%	27	29.7%	91	
	Obese I	1	1.7%	4	6.7%	Total	25.0%	17	28.3%	23	38.3%	60	
	Obese II	1	25.0%	0	0.0%	Total	0.0%	1	25.0%	2	50.0%	4	
	Obese III	0	0.0%	0	0.0%	Total	0.0%	0	0.0%	1	100.0%	1	
	Total	21	8.5%	13	5.3%	Total	22.8%	60	24.4%	96	39.0%	246	

^#^ Fisher’s exact test was used.

**Table 4:** Symptomatic patients undergoing colonoscopy (*n* = 246).

		CRC		Advanced Adenoma		Non-Advanced Adenoma		Others		Normal		Total	*p* Value
		Count	Row %	Count	Row %	Count	Row %	Count	Row %	Count	Row %	Count	
Symptoms	Yes	19	10.3%	11	5.9%	40	21.6%	50	27.0%	65	35.1%	185	0.069 *
	No	2	3.3%	2	3.3%	16	26.2%	10	16.4%	31	50.8%	61	

* The Chi-square test was used.

**Table 5:** Symptoms among patients undergoing colonoscopy (*n* = 246).

		CRC		Advanced Adenoma		Non-Advanced Adenoma		Others		Normal		*p* Value
		Count	Row %	Count	Row %	Count	Row %	Count	Row %	Count	Row %	
PR bleed	yes	11	15.3%	3	4.2%	16	22.2%	30	41.7%	12	16.7%	<0.001 *
	no	10	5.7%	10	5.7%	40	23.0%	30	17.2%	84	48.3%	
Altered Bowel habit	yes	9	21.4%	2	4.8%	9	21.4%	6	14.3%	16	38.1%	0.030 ^#^
	no	12	5.9%	11	5.4%	47	23.0%	54	26.5%	80	39.2%	
Constitutional symptom	yes	5	55.6%	1	11.1%	1	11.1%	1	11.1%	1	11.1%	<0.001 ^#^
	no	16	6.8%	12	5.1%	55	23.2%	59	24.9%	95	40.1%	
Abdominal pain	yes	4	23.5%	2	11.8%	0	0.0%	6	35.3%	5	29.4%	0.006 ^#^
	no	17	7.4%	11	4.8%	56	24.5%	54	23.6%	91	39.7%	
Constipation	yes	1	1.6%	4	6.5%	14	22.6%	8	12.9%	35	56.5%	0.003 *
	no	20	10.9%	9	4.9%	42	22.8%	52	28.3%	61	33.2%	
Anaemia	yes	1	33.3%	1	33.3%	1	33.3%	0	0.0%	0	0.0%	0.022 ^#^
	no	20	8.2%	12	4.9%	55	22.6%	60	24.7%	96	39.5%	
Abdominal mass	yes	0	0.0%	0	0.0%	0	0.0%	0	0.0%	1	100.0%	0.468 ^#^
	no	21	8.6%	13	5.3%	56	22.9%	60	24.5%	95	38.8%	
Tenesmus	yes	0	0.0%	1	14.3%	1	14.3%	3	42.9%	2	28.6%	0.500 ^#^
	no	21	8.8%	12	5.0%	55	23.0%	57	23.8%	94	39.3%	
Perianal abscess	yes	0	0.0%	0	0.0%	0	0.0%	1	50.0%	1	50.0%	1.000 ^#^
	no	21	8.6%	13	5.3%	56	23.0%	59	24.2%	95	38.9%	

* The Chi-square test was used. ^#^ Fisher’s exact test was used.

**Table 6:** Multitarget stool DNA results (*n* = 246).

		CRC		Advanced Adenoma		Non-Advanced Adenoma		Others		Normal		Total	*p* Value
		Count	Row N %	Count	Row N %	Count	Row N %	Count	Row N %	Count	Row N %	Count	
MULTITARGET STOOL DNA	Positive	12	36.4%	3	9.1%	5	15.2%	5	15.2%	8	24.2%	33	<0.001 *
	Negative	7	5.6%	7	5.6%	31	24.6%	32	25.4%	49	38.9%	126	
	Invalid	2	2.3%	3	3.4%	20	23.0%	23	26.4%	39	44.8%	87	
	Total	21	8.5%	13	5.3%	56	22.8%	60	24.4%	96	39.0%	246	

* The Chi-square test was used.

**Table 7:** Multitarget stool DNA (mt-sDNA) for CRC (*n* = 159).

Parameter	CRC	Non-CRC
Positive mt-sDNA	12	21
Negative mt-sDNA	7	119

**Table 8:** Sensitivity, specificity, PPV and NPV of multitarget stool DNA (mt-sDNA) for CRC.

Statistics	Value	95% CI
Sensitivity	63.2%	38.36% to 83.71%
Specificity	85%	77.99% to 90.47%
Positive Predictive Value (PPV)	36.4%	25.30% to 49.08%
Negative Predictive Value (NPV)	94.4%	90.38% to 96.85%

**Table 9:** Multitarget stool DNA (mt-sDNA) for AA (*n* = 140).

Parameter	AA	Non-AA
Positive mt-sDNA	3	18
Negative mt-sDNA	7	112

**Table 10:** Sensitivity, specificity, PPV and NPV of multitarget stool DNA (mt-sDNA) for AA.

Statistics	Value	95% CI
Sensitivity	30.0%	6.67% to 65.25%
Specificity	86.2%	79.00% to 91.58%
Positive Predictive Value (PPV)	14.3%	5.57% to 32.03%
Negative Predictive Value (NPV)	94.1%	91.38% to 96.02%

**Table 11:** Multitarget stool DNA (mt-sDNA) for NAA (*n* = 140).

Parameter	NAA	Non-NAA
Positive mt-sDNA	5	16
Negative mt-sDNA	31	88

**Table 12:** Sensitivity, specificity, PPV and NPV of multitarget stool DNA (mt-sDNA) for NAA.

Statistics	Value	95% CI
Sensitivity	13.9%	4.67% to 29.50%
Specificity	84.6%	76.22% to 90.94%
Positive Predictive Value (PPV)	23.8%	10.98% to 44.20%
Negative Predictive Value (NPV)	74.0%	70.86% to 76.82%

## Data Availability

The datasets used and/or analysed during the current study are available from the corresponding author on request.

## Abbreviations

The following abbreviations are used in this manuscript:

ACTB	Beta-actin (internal control gene)
ACS	American Cancer Society
BMI	Body Mass Index
CRC	Colorectal Cancer
DNA	Deoxyribonucleic Acid
FIT	Faecal Immunochemical Test
FIT-DNA	Faecal Immunochemical Test–DNA
gFOBT	Guaiac Faecal Occult Blood Test
HCTM	Hospital Canselor Tuanku Muhriz
iFOBT	Immunological Faecal Occult Blood Test
MNCR	Malaysia National Cancer Registry
MOH	Ministry of Health
mt-sDNA	Multi-Target Stool DNA
MySCan	Malaysian Study on Cancer Survival
NPV	Negative Predictive Value
PCR	Polymerase Chain Reaction
PPV	Positive Predictive Value
PR	Per Rectal
qMSP	Quantitative Methylation-Specific Polymerase Chain Reaction
RECUKM	Research and Ethics Comitee Universiti Kebangsaan Malaysia
SDC2	Syndecan-2
SEPT9	Septin 9
SPSS	Statistical Package for the Social Sciences
TFPI2	Tissue Factor Pathway Inhibitor 2
UKM	Universiti Kebangsaan Malaysia
USPSTF	United States Preventive Services Task Force
